# Small Molecules Restore Bestrophin 1 Expression and Function of Both Dominant and Recessive Bestrophinopathies in Patient-Derived Retinal Pigment Epithelium

**DOI:** 10.1167/iovs.61.5.28

**Published:** 2020-05-18

**Authors:** Jingshu Liu, Rachel L. Taylor, Richard A. Baines, Lisa Swanton, Sally Freeman, Barbara Corneo, Achchhe Patel, Alan Marmorstein, Travis Knudsen, Graeme C. Black, Forbes Manson

**Affiliations:** ^1^ Division of Evolution and Genomic Sciences, Faculty of Biology, The University of Manchester, Manchester, United Kingdom; ^2^ Division of Neuroscience and Experimental Psychology, Faculty of Biology, Medicine and Health, The University of Manchester, Manchester, United Kingdom; ^3^ Division of Molecular and Cellular Function, Faculty of Biology, Medicine and Health, The University of Manchester, Manchester, United Kingdom; ^4^ Division of Pharmacy & Optometry, Faculty of Biology, Medicine and Health, The University of Manchester, Manchester, United Kingdom; ^5^ Stem Cell Core Facility, Columbia University Medical Centre, New York, New York, United States; ^6^ Department of Ophthalmology, Mayo Clinic, Rochester, Minnesota, United States; ^7^ Manchester Centre for Genomic Medicine, Manchester Academic Health Sciences Centre, Manchester University NHS Foundation Trust, St Mary's Hospital, Manchester, United Kingdom

**Keywords:** bestrophin 1, bestrophinopathies, small molecule, sodium phenylbutyrate (4PBA), functional rescue

## Abstract

**Purpose:**

Bestrophinopathies are a group of untreatable inherited retinal dystrophies caused by mutations in the retinal pigment epithelium (RPE) Cl^−^ channel bestrophin 1. We tested whether sodium phenylbutyrate (4PBA) could rescue the function of mutant bestrophin 1 associated with autosomal dominant and recessive disease. We then sought analogues of 4PBA with increased potency and determined the mode of action for 4PBA and a lead compound 2-naphthoxyacetic acid (2-NOAA). Lastly, we tested if 4PBA and 2-NOAA could functionally rescue bestrophin 1 function in RPE generated from induced pluripotent stem cells (iPSC-RPEs) derived from patients with a dominant or recessive bestrophinopathy.

**Methods:**

Global and plasma membrane expression was determined by Western blot and immunofluorescent microscopy, respectively. The effect of 4PBA and 2-NOAA on transcription was measured by quantitative RT-PCR and the rate of protein turnover by cycloheximide chase and Western blot. Channel function was measured by whole-cell patch clamp.

**Results:**

4PBA and 2-NOAA can rescue the global and membrane expression of mutant bestrophin 1 associated with autosomal dominant disease (Best vitelliform macular dystrophy [BVMD]) and autosome recessive bestrophinopathy (ARB), and these small molecules have different modes of action. Both 4PBA and 2-NOAA significantly increased the channel function of mutant BVMD and ARB bestrophin 1 in HEK293T and iPSC-RPE cells derived from patients with BVMD and ARB. For 4PBA, the increased mutant channel function in BVMD and ARB iPSC-RPE was equal to that of wild-type iPSC-RPE bestrophin 1.

**Conclusions:**

The restoration of bestrophin 1 function in patient-derived RPE confirms the US Food and Drug Administration–approved drug 4PBA as a promising therapeutic treatment for bestrophinopathies.

Bestrophin 1 is a homopentameric Ca^2+^-activated Cl^−^ channel predominantly expressed on the basolateral membrane of the retinal pigment epithelium (RPE), although its physiologic role remains unclear.^1^^–^^3^ Mutations in *BEST1* result in distinct ocular phenotypes of different inheritance patterns collectively referred to as bestrophinopathies, including Best vitelliform macular dystrophy (BVMD, OMIM#153700) and autosomal recessive bestrophinopathy (ARB, OMIM#611809).^4^^–^^7^ More than 250 *BEST1* pathogenic mutations have been identified, most being missense variants associated with BVMD. Most of the mutant bestrophin 1 proteins studied are mislocalized to the cytoplasm, although around one-third correctly traffic to the plasma membrane. All bestrophin 1 mutants studied so far have decreased or absent Cl^−^ current. These data suggest that different disease mechanisms impair function leading to bestrophinopathy phenotypes.

The small molecule sodium phenylbutyrate (4PBA) acts as a chemical chaperone to enhance protein folding in the endoplasmic reticulum (ER), and may also be a histone deacetylase inhibitor (HDACi) that increases stress response gene transcription^8^.^8^ A number of studies have demonstrated that 4PBA can rescue the function of mutant proteins associated with diseases including α1-anti-trypsin deficiency, cystic fibrosis, glaucoma, retinitis pigmentosa, and ARB, implying a general mechanism.^9^^–^^12^

In this study, we show that small molecules are able to rescue the function of mutant bestrophin 1 proteins that cause autosomal dominant (p.Y85H, p.R218C, p.L234V, p.N296S, p.K30R) as well as recessive (p.M325T, p.R255Q) retinal phenotypes. Importantly, we also demonstrate, through the generation of disease-specific patient-derived RPE generated from induced pluripotent stem cells (iPSC-RPEs) (p.K30R, p.R255Q), that such small molecules can functionally rescue mutant bestrophin 1 channel conductivity in human disease-relevant tissue.

Given the cost of gene-specific therapies, compounds that can target multiple dominant and recessive diseases resulting from different mechanistic pathologies are of significant interest.

## Methods

### Cell Culture

MDCKII cell lines were cultured in Dulbecco's modified Eagle's medium (DMEM) containing 10% (v/v) heat-inactivated fetal bovine serum (FBS), 2 mM L-glutamine, and MEM nonessential amino acid. HEK293T cells were cultured in DMEM containing 10% (v/v) FBS and 2 mM L-glutamine. All cells were incubated at 37°C, 5% CO_2_.

### Generation of Stable Cell Lines

The Flp-in T-Rex system (Life Technologies, Paisley, UK) was used to generate stable MDCKII cell lines expressing wild-type, BVMD (p.L234V and p.N296S), and ARB (p.M325T) bestrophin 1. A reverse transfection protocol was used to improve the transfection efficiency. Bestrophin 1 constructs in pcDNA5/FRT/TO were cotransfected with pOG44 into MDCKII Flp-in T-Rex host cells using Lipofectamine LTX and Plus reagent (Life Technologies). Cells were selected with 400 µg/mL hygromycin and 4 µg/mL blasticidin for 2 weeks.

### Generation of iPSCs

iPSCs were derived from the peripheral blood of a patient with ARB who had the homozygous *BEST1* mutation p.R255Q using Sendai virus–mediated gene transfer.^13^ Full details are in the Supplementary Material.

### Differentiation of iPSCs Into Retinal Pigment Epithelium

p.R255Q iPSCs were differentiated to RPE as previously reported^14^.^14^ Full details are in the Supplementary Material. Wild-type iPSC-RPE was purchased from LAgen Laboratories (Rochester, MN, USA). p.K30R iPSC-RPE was a gift from Prof. Marmorstein (Mayo Clinic, Rochester, MN, USA).

### Transient Transfection

HEK293T cells were cotransfected with wild-type or mutant bestrophin 1 and green fluorescent protein (GFP) in a 4:1 ratio using Fugene HD transfection reagent (Promega, Southampton, UK). The transfection medium was replaced with culture medium, with or without treatment, after 24 hours and incubated a further 24 hours before use.

### Small-Molecule Treatment

Compounds were purchased from Sigma-Aldrich (Gillingham, Dorset, UK). Then, 2.5 mM of each was added to cell culture media 24 hours prior to harvesting for Western blot, immunofluorescence fixation, or patch-clamp analysis.

### Real-Time Quantitative RT-PCR

Total RNA was extracted from stably transfected MDCKII cell lines expressing bestrophin 1 using the RNeasy Mini Kit (Qiagen, Manchester, UK). cDNA was generated from 500 ng RNA using the High-Capacity RNA-to-cDNA kit (Applied Biosystems, Loughborough, UK) according to the protocol. The 20-µL quantitative RT-PCR (qRT-PCR) reaction including 1× Power SYBR Green PCR Master Mix (Applied Biosystems), 25 ng DNA, and 0.25 µM forward and reverse primers was conducted at 95°C for 10 minutes followed by 40 cycles of 95°C for 15 seconds and 60°C for 1 minute on a StepOnePlus Real-Time PCR System (Applied Biosystems). Four previously validated reference genes for the quantification of gene expression in the MDCK cell line were tested: ribosomal protein S19 (RPS19), ribosomal protein S5 (RPS5), β-2-microglobulin (B2M), and hypoxanthine phosphoribosyltransferase (HPRT).^15^ Two reference genes gave acceptable efficiencies: *B2M* (96%) and *HPRT* (94%). The *BEST1* primer pair efficiency was 92%. Data were analyzed by the Pfaffl method for multiple reference genes (https://toptipbio.com/qpcr-multiple-reference-genes/). All qRT-PCR was analyzed in biological and technical triplicates.

### Cycloheximide Treatment

Polarized MDCKII cells stably expressing bestrophin 1 were induced with tetracycline, and 4PBA or 2-naphthoxyacetic acid (2-NOAA) was added to the media for 24 hours. The media were then removed and replaced with fresh media containing 20 µg/mL cycloheximide (CHX). Cells were harvested at 0-, 2-, 4-, 6-, and 8-hour time points before Western blotting.

### SDS-PAGE and Western Blot

Cells were lysed in RIPA buffer and lysates were loaded onto a 12% Mini PROTEAN TGX Stain-Free Gel (Bio-Rad, Watford, UK). Gels were transferred onto nitrocellulose membrane (LI-COR Biosciences, Cambridge, UK) by wet transfer overnight at 4°C. The membrane was blocked in 5% milk in TBS-T (Tris-buffer saline, 0.1% Tween 20) at room temperature (RT) for 1 hour and then incubated with primary antibodies in 2% milk TBS-T for 1 hour at RT. After three washes in TBS-T, membranes were incubated with secondary antibodies in 2% milk TBS-T for 1 hour at RT and then washed three times in TBS-T before imaging. Antibodies are listed in Supplementary Table S1. An anti–β-actin antibody was used as loading control. The LI-COR Odyssey CLx system was used to visualize the membrane and LI-COR Image Studio 5.0 to analyze the image.

### Immunofluorescence

Stable MDCKII cells were polarized on glass coverslips for 7 days and induced with tetracycline for 24 hours before fixing with 4% paraformaldehyde for 15 minutes at RT. Cells were permeabilized with PBST (0.2% Tween 20 in PBS) for 20 minutes at RT and blocked with 10% serum (v/v in 0.05% Tween 20) for 30 minutes at RT. The primary antibody in 2% serum was added overnight at 4°C before the cells were washed three times in PBST and incubated in the secondary antibodies (diluted in 0.05% Tween 20) for 30 minutes at RT (antibodies listed in Supplementary Table S1). Cells were then washed three times in PBS followed by one wash in distilled water. Coverslips were air dried before mounting with Prolong Gold antifade reagent with 4′,6-diamidino-2-phenylindole (DAPI) (Thermo Fisher Scientific, Altringham, UK). Cells were imaged on a Zeiss Axioplan2 upright microscope (Carl Zeiss, Oberkochen, Germany) using a 63× objective and captured using an Axiocam MrM camera (Carl Zeiss) through Aixovision v4.8.2 software (Carl Zeiss). Specific band pass filter sets for DAPI, FITC, and Texas red were used to prevent channel bleed through. Images were processed with Fiji^16^.^16^ For quantitative analysis, the membrane area was selected after subtracting background, and the average bestrophin 1 signal within the defined membrane area was calculated by the “ROI manager” tool in Fiji.

### Whole-Cell Patch Clamp

Transfected HEK293T cells or iPSC-RPE cells were dissociated and seeded on poly-L-lysine (HEK293T) or Matrigel (Corning, New York, NY, USA) (iPSC-RPE) coated 13-mm glass coverslips for 24 hours (HEK293T) or 24- to 72-hour (iPSC-RPE) incubation before analysis. Whole-cell patch clamp was performed as previously described^12^.^12^

### Data Analysis

Quantitative data were collected from at least three independent experiments and shown in graphs as mean ± SEM. Statistical analysis was performed by Student's *t*-test or 1-way ANOVA by GraphPad Prism 7.0 (GraphPad Software, La Jolla, CA, USA), and statistically significant difference was defined as *P* < 0.05.

## Results

### 4PBA Increases the Expression of Dominant BVMD Bestrophin 1

**Figure 1.**
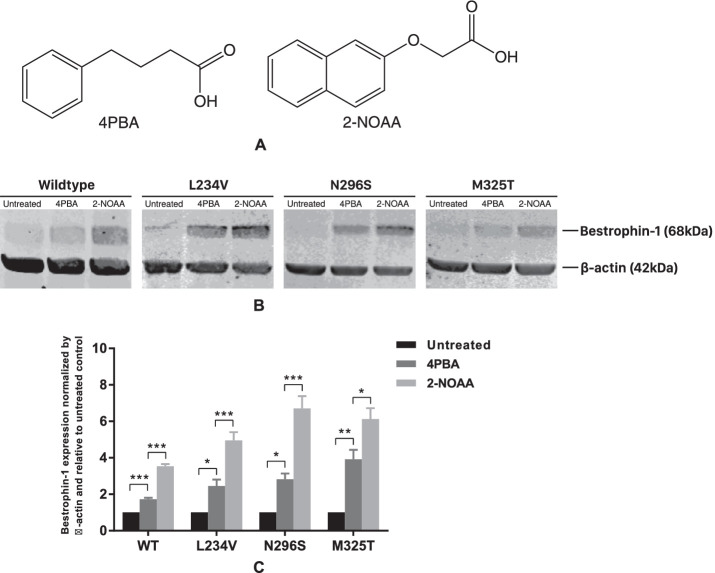
Effect of 4PBA and 2-NOAA on wild-type, BVMD, and ARB mutant bestrophin 1 expression in transfected MDCKII cells. (**A**) Chemical structures of 4PBA and 2-NOAA. (**B**) Western blots of stably transfected MDCKII cells expressing wild-type, BVMD (p.L234V and p.N296S), or ARB (p.M325T) mutant bestrophin 1 treated with 4PBA and 2-NOAA. (**C**) Quantification of Western blot data from at least four independent experiments is presented as mean ± SEM. *P < 0.05, **P < 0.01, and ***P < 0.001 indicate significant differences between the indicated treatments as determined by 1-way ANOVA. The expression of bestrophin 1 is normalized to the loading control (β-actin) and relative to untreated group that is taken as 1 in the graph.

The small molecule 4PBA has previously been shown to increase the expression of wild-type and ARB mutant bestrophin 1 (Fig. 1A)^12^.^12^ We therefore tested whether 4PBA has a similar effect on the global expression of missense mutant bestrophin 1 proteins associated with BVMD (p.L234V and p.N296S). 4PBA was used at 2.5 mM and was added to cell cultures 24 hours before harvesting and the lysate subjected to Western blot analysis. For both BVMD missense mutations and an ARB-positive control (p.M325T), 4PBA significantly increased the global expression of the mutant bestrophin 1 proteins (Figs. 1B, 1C). As before, 4PBA also significantly increased the expression of wild-type bestrophin 1 (Figs. 1B, 1C).

### Testing for 4PBA Analogues That Increase Mutant Bestrophin 1 Expression

The concentration of 4PBA used was 2.5 mM, equivalent to nearly the maximum tolerated dose for humans. In addition, the daily dose of 25 to 30 g 4PBA required for therapeutic effect is a large amount to ingest in a tablet form. This prompted us to investigate whether other small molecules were more potent. We tested seven compounds that have similar chemical structures to 4PBA for their effect on the global expression of missense mutant bestrophin 1 proteins associated with either BVMD (p.L234V and p.N296S) or ARB (p.M325T) in stably transfected MDCKII cells (Supplementary Fig. S1A).

All test compounds were used at 2.5 mM and added to cell cultures 24 hours before harvesting and the lysate subjected to Western blot analysis. Notably, the 4PBA analogue 2-NOAA significantly increased the expression of each mutant bestrophin 1 protein above that of 4PBA (Figs. 1A, 1B, 1C). None of the other six compounds had an obvious effect on mutant bestrophin 1 expression (Supplementary Fig. S1B). Like 4PBA, 2-NOAA also significantly increased the expression of wild-type bestrophin 1 (Figs. 1B, 1C).

### 4PBA and 2-NOAA Increase the Expression of Dominant and Recessive Mutant Bestrophin 1 at the Plasma Membrane

**Figure 2.**
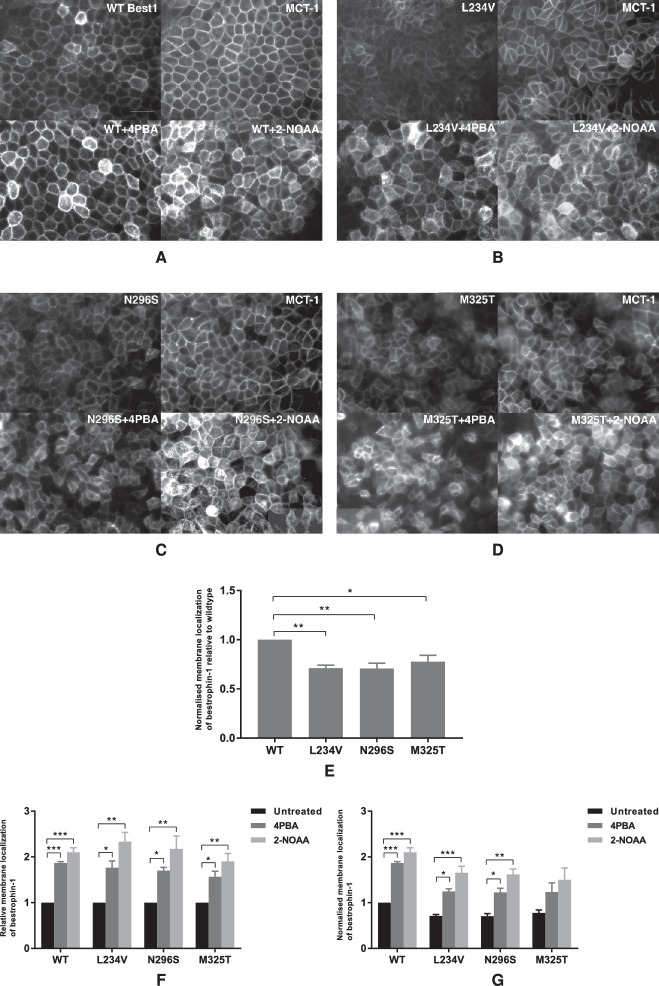
Localization of bestrophin 1 and basolateral membrane marker MCT-1 in stable MDCKII cells expressing (**A**) wild-type, (**B**) BVMD p.L234V, (**C**) BVMD p.N296S, or (**D**) ARB p.M325T bestrophin 1 with or without treatment. (**E–G**) Quantification of plasma membrane localization was calculated from three independent experiments and is presented as mean ± SEM. *P < 0.05, **P < 0.01, and ***P < 0.001 indicate significant differences between untreated and treated groups. (**E**) Normalized membrane expression of mutant bestrophin 1 compared to wild-type. (**F**) Expression level of membrane-localized bestrophin 1 normalized to the untreated control. (**G**) Expression level of membrane-localized bestrophin 1 normalized to untreated wild-type bestrophin 1. *Scale bar*: 20 µm.

We sought to determine whether the increased expression levels of dominant and recessive mutant bestrophin 1 proteins correlated with their increased expression at the plasma membrane. To do this, we used MDCKII cells, an epithelial cell line that has been extensively used by a number of research groups as a model for RPE. The cells achieve a clear apicobasal polarization 7 days after reaching confluence. The cellular localization of bestrophin 1 was determined by immunofluorescence using monocarboxylate transporter 1 (MCT-1) as a marker for the basolateral plasma membrane. Membrane quantification was determined by quantifying the average bestrophin 1 signal at the plasma membrane using software analysis of digital images. Wild-type bestrophin 1 protein colocalized with MCT-1 and correctly localized to the plasma membrane (Fig. 2A). Each of the three mutant bestrophin 1 proteins, whether associated with dominant BVMD or recessive ARB, also had a predominant plasma membrane localization without treatment, although at a significantly lower level compared to the wild-type protein (Fig. 2E). After treatment with either 4PBA or 2-NOAA, the plasma membrane-localized bestrophin 1 was significantly increased for both wild-type and mutant bestrophin 1 proteins (Fig. 2F). There was no significant difference in increased plasma membrane localization between 4PBA and 2-NOAA. For the two mutant bestrophin 1 proteins associated with BVMD (p.L234V and p.N296S), treatment with either 4PBA or 2-NOAA significantly increased plasma membrane localization to that of wild-type bestrophin 1 (Fig. 2G). There was no significant difference in plasma membrane localization between untreated, 4PBA-treated, or 2-NOAA–treated p.M325T bestrophin 1 and wild-type bestrophin 1. These data show that wild-type and mutant bestrophin 1 treated with either 4PBA or 2-NOAA results in their increased localization to the plasma membrane.

### Different Action of 4PBA and 2-NOAA on Bestrophin 1 Synthesis and Stability

**Figure 3.**
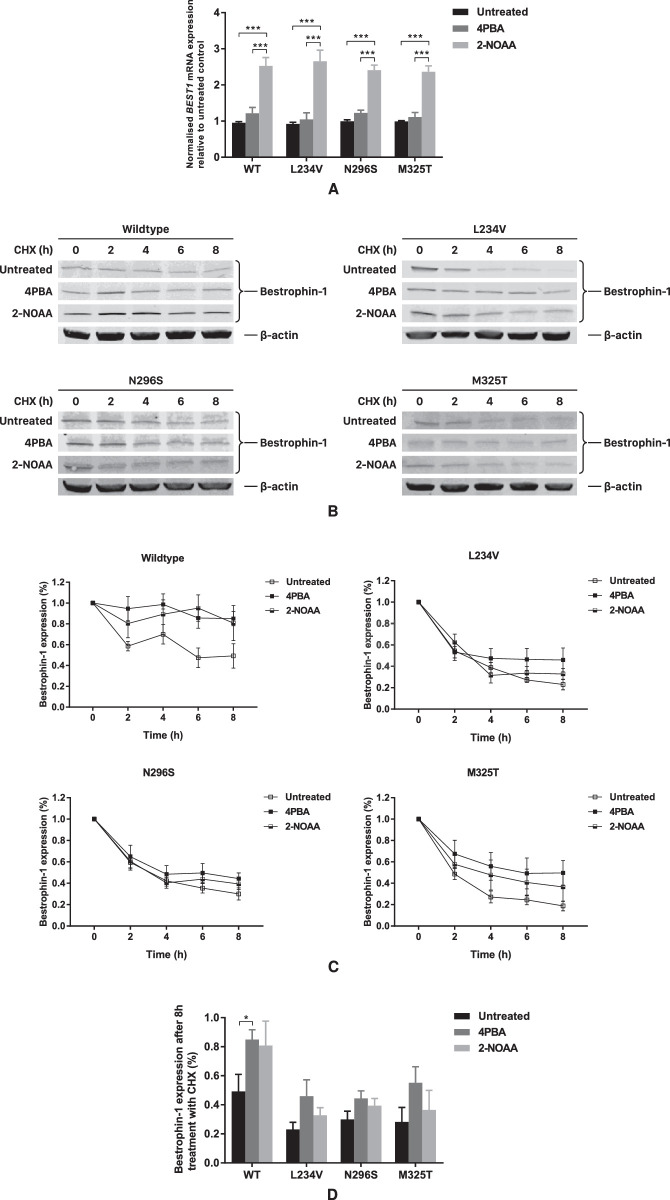
Effect of 4PBA and 2-NOAA on *BEST1* transcription and protein turnover. (**A**) *BEST1* mRNA level in MDCKII cells stably expressing wild-type or mutant bestrophin 1 with or without 4PBA and 2-NOAA treatment. Quantification of qRT-PCR data from three independent experiments is presented as mean ± SEM. ***P < 0.001 indicates significant differences between the indicated treatments as determined by 1-way ANOVA. For each cell line, the expression level of *BEST1* mRNA is normalized to reference genes *B2M* and *HPRT* and relative to the untreated control. (**B**) Western blot results of stable MDCKII cells treated by CHX for 0 to 8 hours. Cells were untreated or grown in the presence of 4PBA or 2-NOAA for 24 hours before the media were replaced with fresh media containing only CHX. (**C**) Plots of quantified Western blot data from at least three independent experiments are presented as mean ± SEM. (**D**) Bar chart of bestrophin 1 levels after 8 hours of CHX treatment following no treatment or the addition of 4PBA. The expression of bestrophin 1 is normalized to the loading control (β-actin). Significance was determined by Student's *t*-test. *P < 0.05 indicates significant difference between treated and untreated cells.

The mode of action of 4PBA is not fully understood, and it may act in more than one way (e.g., chemical chaperone, HDACi). It is likely the effect is cellular rather than protein specific as a number of mutant proteins show increased expression and function in its presence. We investigated whether 4PBA and 2-NOAA increased bestrophin 1 expression by increasing protein synthesis or decreasing protein degradation (increasing protein stability). We performed qRT-PCR on stable MDCKII cell lines and found that 4PBA had no significant effect on *BEST1* transcription. Surprisingly, its analogue, 2-NOAA, significantly increased the expression of *BEST1* mRNA, showing that these two structurally similar small molecules increase protein expression by different mechanisms (Fig. 3A). This hypothesis was confirmed by blocking translation with CHX after adding the test compounds. MDCKII cells stably expressing wild-type or mutant bestrophin 1 were treated with 4PBA or 2-NOAA for 24 hours before adding CHX for up to 8 hours before harvesting. Cell lysates were then analyzed by Western blot. BVMD or ARB mutant bestrophin 1 underwent a faster rate of degradation compared to wild-type bestrophin 1 protein, suggesting the mutant bestrophin 1 proteins are less stable than wild-type (Fig. 3C). Treating cell cultures with 4PBA before removing it and adding CHX resulted in more wild-type and mutant bestrophin 1 remaining after 8 hours compared to cells grown in the absence of 4PBA, although this was only significant for the wild-type protein (Figs. 3B, 3C, 3D). Treating cell cultures with 2-NOAA prior to removing it and adding CHX had a similar but reduced effect on wild-type and mutant bestrophin 1 degradation compared to 4PBA. None of these increases were significant (Figs. 3B, 3C, 3D). Using nonlinear regression (exponential function) to the data in Fig. 2C enabled an estimation of the effect of 4PBA and 2-NOAA on the half-life of wild-type and mutant bestrophin 1 (Table). Taken together with the qRT-PCR data, these results indicate that 4PBA and 2-NOAA act by different mechanisms, with 2-NOAA acting to increase gene expression whereas 4PBA does not. The data show that 4PBA has a small effect on slowing the rate of mutant protein turnover, but this is insignificant.

**Table.** Half-Life (Hours) of Wild-Type and Mutant Bestrophin 1

Characteristic	Untreated	4PBA	2-NOAA
Wild-type	7.4	32.4	43.6
L234V	3.1	5.9	3.6
N296S	3.9	5.9	4.8
M325T	2.5	6.7	4.7

### 4PBA and 2-NOAA Rescue the Channel Function of Heterologously Expressed BVMD and ARB Bestrophin 1

Having shown that 4PBA and 2-NOAA both increase the global and membrane expression of dominant and recessive mutant bestrophin 1 proteins, we tested whether the increased plasma membrane expression correlated to increased Cl^−^ conductance using whole-cell patch clamp. HEK293T cells were transiently transfected with either wild-type or mutant bestrophin 1 (p.L234V, p.N296S, or p.M325T) and GFP for the identification of transfected cells for patch clamp. Whole-cell patch clamp is the electrophysiologic gold standard for measuring ion current resulting exclusively from channels at the plasma membrane. A reduced or abolished Cl^−^ conductance is characteristic of all BVMD and ARB mutant bestrophin 1 proteins studied.

**Figure 4.**
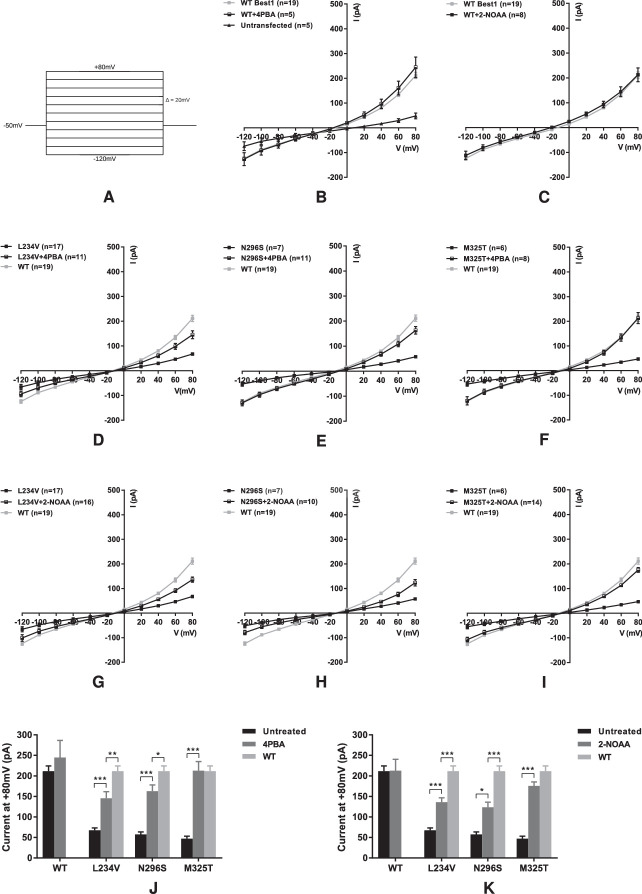
Current-voltage (I-V) relationships in HEK293T cells expressing wild-type bestrophin 1, BVMD mutants p.L234V and p.N296S or ARB mutant p.M325T with or without 4PBA (**B**, **D**, **E**, and **F**, respectively) or 2-NOAA (**C**, **G**, **H**, and **I**, respectively) treatment. Recordings were taken from –120 mV to +80 mV in Δ20-mV steps of 2 seconds each. The voltage protocol is shown in (**A**). Current of untransfected HEK293T cell is shown in (**B**). (**J**) Currents at +80 mV for wild-type and mutant bestrophin 1 proteins with or without 2.5 mM 4PBA. (**K**) Currents at +80 mV for wild-type and mutant bestrophin-1 proteins with or without 2.5 mM 2-NOAA. Data are presented as mean ± SEM. *P < 0.05, **P < 0.01, and ***P < 0.001 indicate significant differences between groups. *n* = number of cells patched.

4PBA or 2-NOAA, each at 2.5 mM, was added to the culture media of HEK293T cells transiently expressing wild-type or mutant bestrophin 1 24 hours before performing whole-cell patch clamp. The current-voltage relationships and currents at +80 mV of each group are shown in Figure 4. Both 4PBA and 2-NOAA significantly increased the Cl^−^ conductance of both BVMD and ARB mutants, and for the ARB p.M325T mutant, the Cl^−^ conductance was fully restored to that of the wild-type protein, consistent with previous findings (Figs. 4D–4K)^12^.^12^ The Cl^−^ currents for both the BVMD mutants (p.L234V and p.N296S) were significantly increased with both small-molecule treatments compared to untreated (*P* < 0.001). 4PBA increased the conductance of p.L234V BVMD bestrophin 1 from 32% to 69% of the wild-type current and 2-NOAA from 32% to 64%. The situation was similar for the second BVMD mutant p.N296S; 4PBA increased the current from 22% to 77% of the wild-type current and 2-NOAA from 22% to 58%, although the increased current remained below that of the wild-type (*P* < 0.05) (Figs. 4J, 4K). Neither 4PBA nor 2-NOAA had a significant effect on the Cl^−^ conductance of wild-type bestrophin 1 (Figs. 4B, 4C, 4J, 4K).

**Figure 5.**
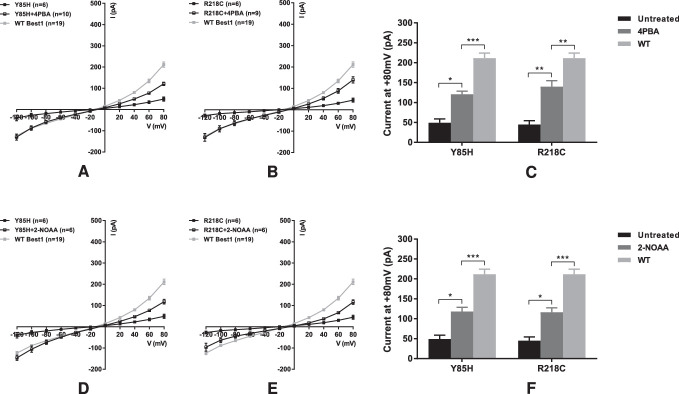
Current-voltage (I-V) relationships in HEK293T cells expressing BVMD p.Y85H or p.R218C bestrophin 1 with or without 4PBA (**A**, **B**, respectively) or 2-NOAA treatment (**D**, **E**, respectively). (**C**) Currents at +80 mV of wild-type and each mutant with or without 4PBA treatment. (**F**) Currents at +80 mV of wild-type and each mutant with or without 2-NOAA treatment. Data are presented as mean ± SEM. *P < 0.05, **P < 0.01, and ***P < 0.001 indicate significant differences between groups. *n* = number of cells patched.

To further demonstrate the rescue effect of 4PBA and 2-NOAA on the channel function of BVMD mutant bestrophin 1, we tested an additional two mutations, p.Y85H and p.R218C, both of which are recurrent mutations reported in more than eight pedigrees. As with p.L234V and p.N296S, 4PBA and 2-NOAA both significantly increased the Cl^−^ current for these mutant proteins (p.Y85H: + 4PBA 23% to 57% of wild-type, + 2-NOAA 23% to 56%; p.R218C: + 4PBA 21% to 66% of wild-type, + 2-NOAA 21% to 55%) (Fig. 5). The electrophysiological data show that 4PBA and 2-NOAA both significantly increase the channel function of all tested BVMD mutant bestrophin 1 proteins, and both fully restore the activity of the ARB mutant protein.

### Small Molecules Rescue the Function of BVMD and ARB Mutant Bestrophin 1 in Patient-Derived RPE

**Figure 6.**
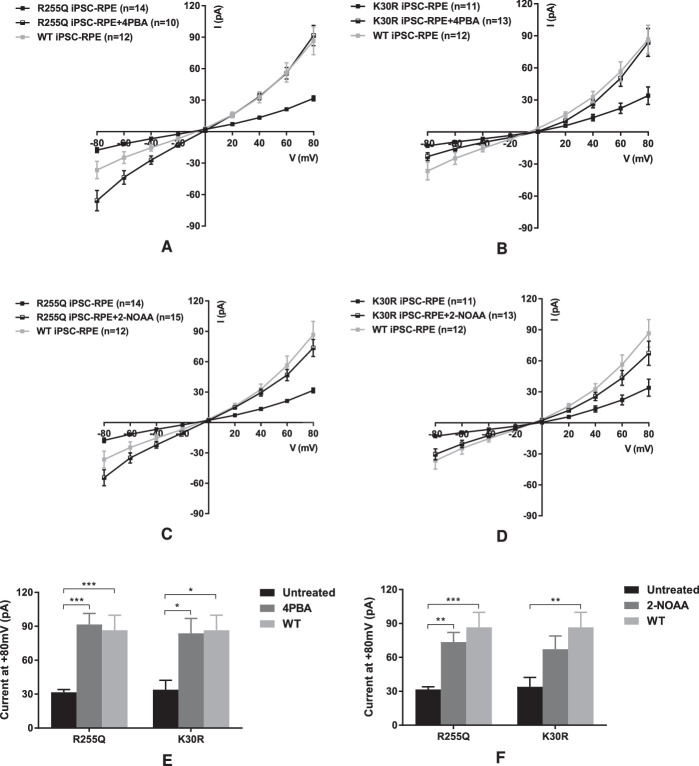
Current-voltage (I-V) relationships in p.K30R, p.R255Q, and wild-type iPSC-RPE cells with or without 4PBA (**A**, **B**, respectively) or 2-NOAA (**C**, **D**, respectively) treatment. (**E**) Currents at +80 mV from mutant iPSC-RPE cells with or without 4PBA treatment. (**F**) Currents at +80 mV from mutant iPSC-RPE cells with or without 2-NOAA treatment. Data are presented as mean ± SEM. *P < 0.05, **P < 0.01, and ***P < 0.001 indicate significant differences between groups. *n* = number of cells patched.

To confirm the functional rescue in HEK293T cells was also relevant in the endogenous tissue type, we generated RPE from patient iPSCs harboring either a homozygous ARB p.R255Q mutation or a heterozygous p.K30R BVMD mutation (Supplementary Fig. S4). Treatment with 4PBA and 2-NOAA fully restored the Cl^−^ current to p.R255Q iPSC-RPE cells, equal to that recorded from iPSC-RPE generated from a normal unrelated donor with no bestrophin 1 mutation (Figs. 6A, 6C, 6E, 6F). Both small molecules also increased the Cl^−^ current recorded from the p.K30R BVMD RPE cells, and for 4PBA, this increase was also significant (Figs. 6B, 6D, 6E, 6F).

## Discussion

Protein misfolding and instability caused by an altered peptide sequence is an important pathologic mechanism in Mendelian disorders. The cellular response to such mutant proteins can result in reduced protein expression and mislocalization. Mutant proteins may have reduced activity compared to wild-type, adopt novel functions, or act in a dominant-negative manner. In this study, we first demonstrated that 4PBA, a well-established chemical chaperone, could restore the function in the same way to mutant bestrophin 1 causing an autosomal dominant bestrophinopathy as it can to a recessive one. We next sought more potent small molecules by testing 4PBA analogues and identified 2-NOAA to have similar biological activity to that of 4PBA. We showed that 4PBA and 2-NOAA restore function to mutant proteins associated with autosomal dominant and recessive disease and that they act in different manners. Both compounds were active in patient-derived RPE.

Our exemplar protein, bestrophin 1, is a Cl^−^ channel that is mutated in autosomal dominant and recessive bestrophinopathies. There is no treatment for either of these orphan diseases, a situation mirroring the majority of other genetic diseases associated with unstable or misfolded proteins. Previous studies have shown that ARB-associated mutant proteins are predominantly degraded by the proteasome and that their function can be increased by the addition of 4PBA.^12^^,^^17^ A subsequent study of BVMD and ARB mutant proteins confirmed this, while BVMD mutant proteins were subjected to post-ER degradation via the endolysosomal pathway^18^.^18^ In contrast to the data presented here, the authors found that 4PBA could not rescue the expression of two BVMD mutant bestrophin 1 proteins, although the functional outcomes were not investigated. Notably the p.T6P and p.W93C BVMD mutants caused ER retention and mislocalization, in contrast to the BVMD mutants studied here that localized correctly at the plasma membrane^18^.^18^ This supports the supposition that different mutations causing the same phenotype can have varied impacts at the protein level. Mutant proteins that escape from the ER protein quality control system may have less disruption to their tertiary or quaternary structure and therefore retain some activity, as has been reported for cystic fibrosis transmembrane conductance regulator (CFTR)^19^.^19^ A study testing the CFTR potentiator ivacaftor found highly variable responses to the drug that were related to the severity of the CFTR processing defect^20^.^20^

4PBA is a drug approved by the US Food and Drug Administration (FDA) for the treatment of urea cycle disorders^21^.^21^ The small-molecule drug is also thought to act as a chemical chaperone by assisting protein folding and decreasing ER stress and may also induce the expression of cellular chaperones as an HDACi.^8^^,^^22^ We found that 4PBA increased the amount of mutant bestrophin 1 proteins without significantly affecting gene expression and that bestrophin 1 proteins in the presence of 4PBA had a slower turnover, although the effect was insignificant. Thus, both “quantity and quality” of mutant bestrophin 1 were improved by 4PBA, resulting in membrane expression and channel function that was comparable to that of wild-type.

Among the compounds tested, only 2-NOAA showed a superior effect to 4PBA in increasing global expression of bestrophin 1. By qRT-PCR and CHX experiments, we found that, unlike 4PBA, 2-NOAA increased transcription of *BEST1* mRNA rather than decreasing protein turnover. We suggest that 4PBA acts as a chemical chaperone (or via HDACi) to assist mutant bestrophin 1 proteins to achieve a relatively native structure in the ER that permits their trafficking to the plasma membrane. Neither 4PBA nor 2-NOAA reduced the expression of the ER stress marker GRP78 in MDCKII cell expression wild-type or mutant bestrophin 1 (Supplementary Fig. S3). This is in contrast to a number of previous studies that show 4PBA reduces ER stress. The alternative explanation is that the three mutant proteins tested here did not induce ER stress, as has been shown in other mutant bestrophin 1–expressed MDCKII cell lines before^18^.^18^ In contrast, 2-NOAA upregulates *BEST1* transcription. Both mechanisms increase the Cl^−^ conductance of cells expressing mutant bestrophin 1. Different modes of action between analogues have also been reported for 4PBA and its analogue butyrate. Butyrate, a known transcriptional regulator, increased ∆F508-CFTR transcription and expression but had a mild effect on mutant CFTR function, while 4PBA enhanced the expression and function of the mutant CFTR protein with no effect on transcription.^23^^,^^24^

4PBA and 2-NOAA both increase the activity of mutant bestrophin 1, although the effect is less pronounced for BVMD than ARB mutants. The BVMD-mutated residues in this study (Y85, R218, L234, and N296) are all at functionally critical sites, supporting the notion that they may be intrinsically less active and more difficult to rescue compared to bestrophin 1 associated with ARB mutations^12^.^12^

Wild-type bestrophin 1 normally undergoes some degree of proteasomal degradation, which can be reduced by proteasome inhibitors and 4PBA.^12^^,^^25^ We found the 4PBA-induced increase in the plasma membrane expression of wild-type bestrophin 1 was not accompanied by an increase in the cells’ Cl^−^ current. It may be that cells reach a maximum Cl^−^ conductance beyond which an increase in the number of channels does not correlate with an increase in conductance.

By demonstrating the efficacy of 4PBA in restoring expression and function of mutant bestrophin 1 proteins associated with either BVMD or ARB, we have shown that autosomal dominant and recessive disorders may be amenable to treatment with the same therapeutic molecule. We have shown for the first time, to our knowledge, that a 4PBA analogue is equally effective in correcting the expression and function of autosomal dominant and recessive mutant proteins, confirming the principle for screening analogues of existing bioactive small molecules for novel lead compounds.

The successful restoration of bestrophin 1 function in patient-derived RPE confirms 4PBA as a promising therapeutic treatment for bestrophinopathies and validates the data from cell models used in compound screening. Testing the efficacy of 4PBA on additional mutant bestrophin 1 proteins to the seven investigated here is necessary to confirm the putative utility of 4PBA as a therapeutic treatment for bestrophinopathies, as are in vivo studies to show the in vitro action of 4PBA is consistent. 4PBA could be administered systemically as the sclera and cornea are practically impervious to topical solutions. The normal intraocular pressure of a mouse model of primary open-angle glaucoma was restored using 4PBA eye drops, but it was likely that systemic dosing occurred through the animals licking their eyes^26^.^26^ A concern would be that if 4PBA works by releasing proteins from the ER, the release of undesirable proteins would result in side effects. No obvious side effects to such a mode of action have been reported in previous clinical trials or in urea cycle disorder patients using 4PBA on label (https://www.accessdata.fda.gov/drugsatfda_docs/label/2009/020572s016,020573s015lbl.pdf).^27^^,^^28^

Gene therapy has enormous potential for treating inherited retinal dystrophies, with Luxturna recently achieving FDA approval for Leber congenital amaurosis. However, such approaches are costly and time-consuming to develop and are only applicable to recessive disorders, potentially restricting their application^29^.^29^ The development of alternative therapeutic approaches or adjunctive therapies is essential. The large number of disease proteins amenable to functional correction by small molecules such as 4PBA further strengthens the view that identifying more efficacious molecules is a worthwhile future approach.

## Supplementary Material

Supplement 1
