# Supplementary material for: Small Molecules Restore Bestrophin 1 Expression and Function of Both Dominant and Recessive Bestrophinopathies in Patient-Derived Retinal Pigment Epithelium
Source: Invest Ophthalmol Vis Sci. 2020 May 18;61(5):28. doi: 10.1167/iovs.61.5.28 (PMC7405785; doi:10.1167/iovs.61.5.28)
Supplement: Supplement 1 [file iovs-61-5-28_s001.pdf]

Supplemental Material

Supplementary Methods

Generation of induced pluripotent stem cell (iPSC)

Peripheral blood of an ARB patient with the homozygous *BEST1* mutation p.R255Q was collected in to a BD Vacutainer® Cell Preparation Tube™ with Sodium Citrate (Becton Dickson, USA) and mononuclear cells were isolated according to manufacturers' instructions. Peripheral blood mononuclear cells (PBMCs) were cultured in a media that favoured the expansion of the erythroblast (EB) population as previously described [1]. The EB-enriched cell population was reprogrammed using the CytoTune™-iPS 2.0 Sendai Reprogramming Kit (Thermo Fisher Scientific), according to the manufacturer's protocol with MOI of 5:5:3 (KOS:hc-MYC:hKLF4). Three colonies were manually picked and expanded on irradiated mouse embryonic fibroblasts in hES medium (DMEM/F12, 20% KOSR, 1% NEAA, 1% Penicillin/Streptomycin, 1% L-Glutamine, 0.1% β-mercaptoethanol, 4ng/ml bFGF) (Thermo Fisher Scientific). iPSC clones were characterized for expression of pluripotency markers Alexafluor 488 NANOG (BD Pharmingen), Alexa Fluor 488 OCT4 (Cell Signalling), PerCP-Cy5.5 Tra-1-60 (BD Pharmingen) and Alexa Fluor 488 SSEA4 (BD Pharmingen) by flow cytometry. Data acquisition was conducted on an S3e Cell Sorter (Biorad) and analysis performed using FlowJo (v10.5.1, FlowJo LLC). Pluripotency was assessed using the Human Pluripotency Stem Cell Functional Identification Kit (R&D Systems) according to the manufacturer's protocol. At passage 6, iPSC clones were sent to Cell Line Genetics for verification that a normal karyotype was present.

Differentiation of iPSC into Retinal Pigment Epithelium (RPE)

For p.R255Q, passage 7 iPSCs were transitioned to serum-free, feeder-free conditions by passaging on to culture vessels coated with hESC-qualified Matrigel (Corning) in mTeSR1

medium (Stem Cell Technologies) in a humidified incubator ,at 37℃, 5% CO_2 atmosphere. iPSCs were passaged at least twice using EDTA (Life Technologies), under feeder-free conditions prior to the commencement of the differentiation protocol. Directed differentiation of iPSC-RPE was performed as previously described, up to D18 differentiation [2]. From D18 cells were maintained in RPE-medium with 10mM nicotinamide (Sigma). When a significant number of pigmented cells with RPE morphology had appeared (around D30-40), non-RPE cells were removed by incubation in 1:1 trypsin-EDTA (0.05%, Life Technologies): Collagenase IV (10%, Sigma) for 10-15 min followed by gentle agitation of the monolayer. The remaining adherent RPE cells were detached using dissociation reagent (1:1 Accutase (Innovative Cell Technologies):trypsin-EDTA (0.25%, Life Technologies) supplemented with 12ng/mL DNase (Sigma)), collected by centrifugation and re-plated on Matrigel in RPE-medium supplemented with 10% heat-inactivated FBS (Life Technologies) and 0.5µL/mL Y-27632 dihydrochloride (10mM, R&D Systems). After 24h, the media was replaced with RPE-medium supplemented with 10% heat-inactivated FBS. Prior to patch-clamp analysis, iPSC-RPE cells were detached using dissociation reagent and were passed through a 70µM cell strainer, counted and seeded on Matrigel coated glass coverslips at 2-3x10^5 cells/well of a 12-well plate in RPE-medium supplemented with 10% heat-inactivated FBS and 0.5µL/mL Y-27632 dihydrochloride for 24h. The media was switched to RPE-medium supplemented with 10% heat-inactivated FBS for the remaining culture period. Wildtype iPSC-RPE was purchased from LAgen Laboratories (USA). p.K30R iPSC-RPE was a kind gift from Prof. Alan Marmorstein (Mayo Clinic, Rochester, USA).

Supplementary Figures

A

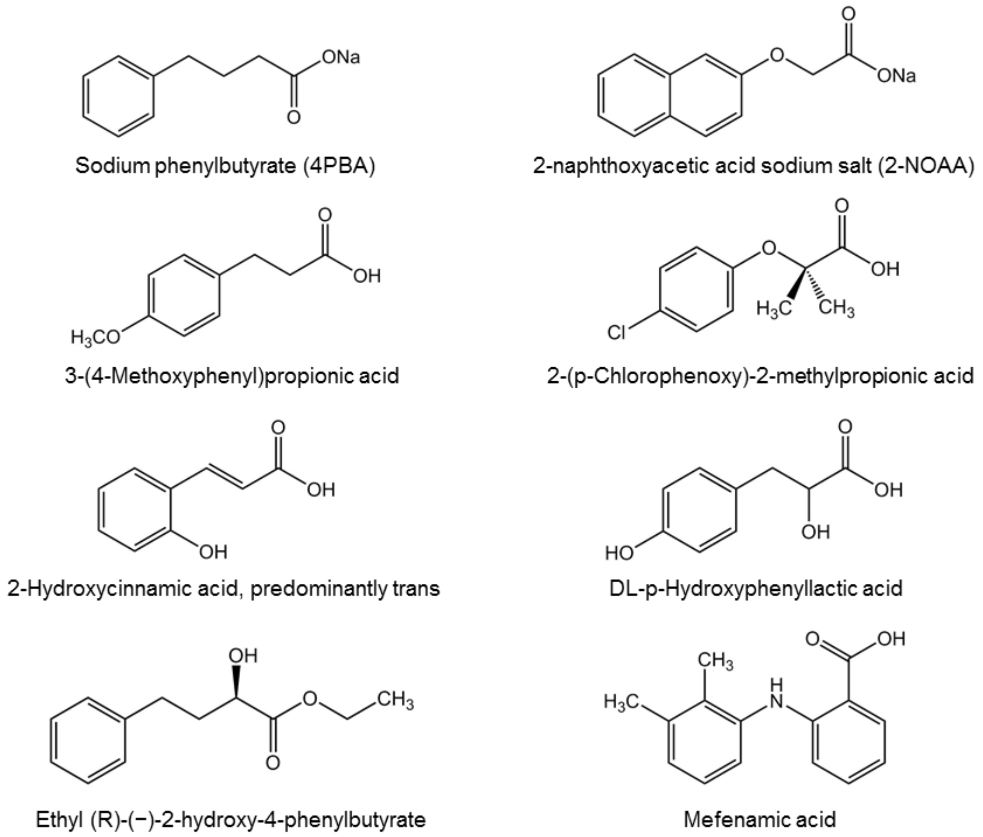

B

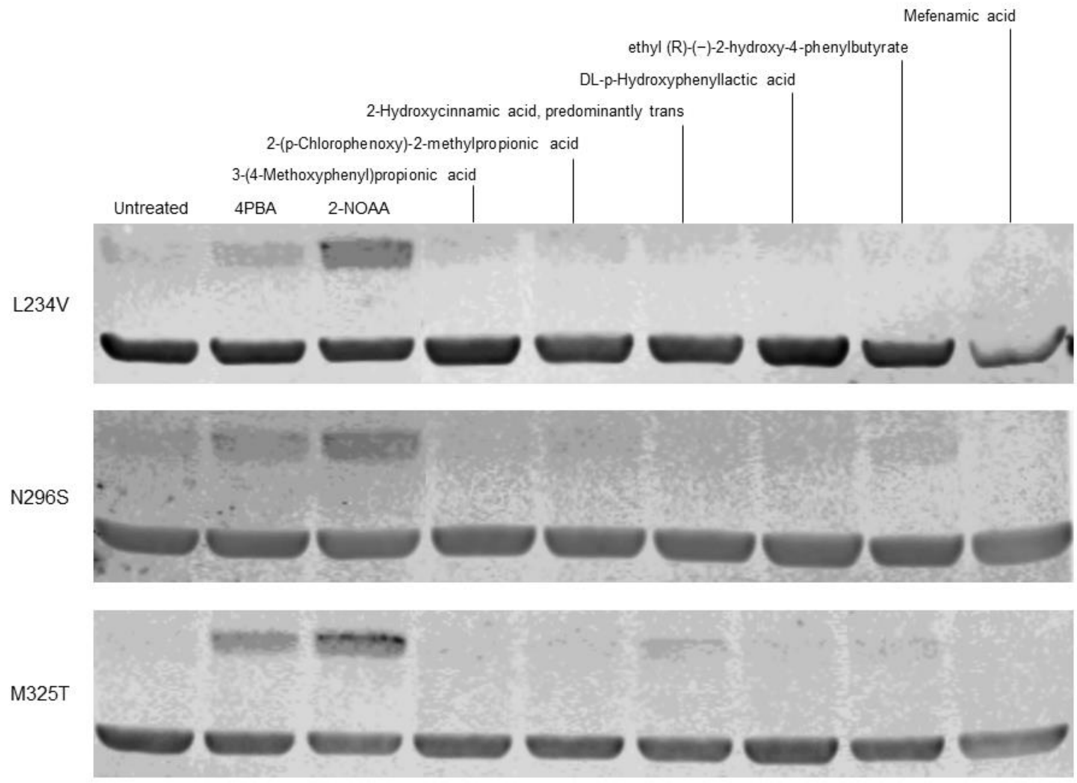

Fig. S1. Compounds screened in this study. (**A**) Chemical structures of 4PBA and related compounds: (1) Sodium phenylbutyrate (4PBA); (2) 2-naphthoxyacetic acid sodium salt (2-NOAA); (3) 3-(4-Methoxyphenyl)propionic acid; (4) 2-(p-Chlorophenoxy)-2-methylpropionic acid; (5) 2-Hydroxycinnamic acid, predominantly trans; (6) DL-p-Hydroxyphenyllactic acid; (7) ethyl (R)-(−)-2-hydroxy-4-phenylbutyrate; (8) Mefenamic acid. (**B**) Western blots of stably transfected MDCKII cells expressing BVMD (p.L234V and p.N296S) or ARB (p.M325T) mutant bestrophin-1 treated with eight compounds.

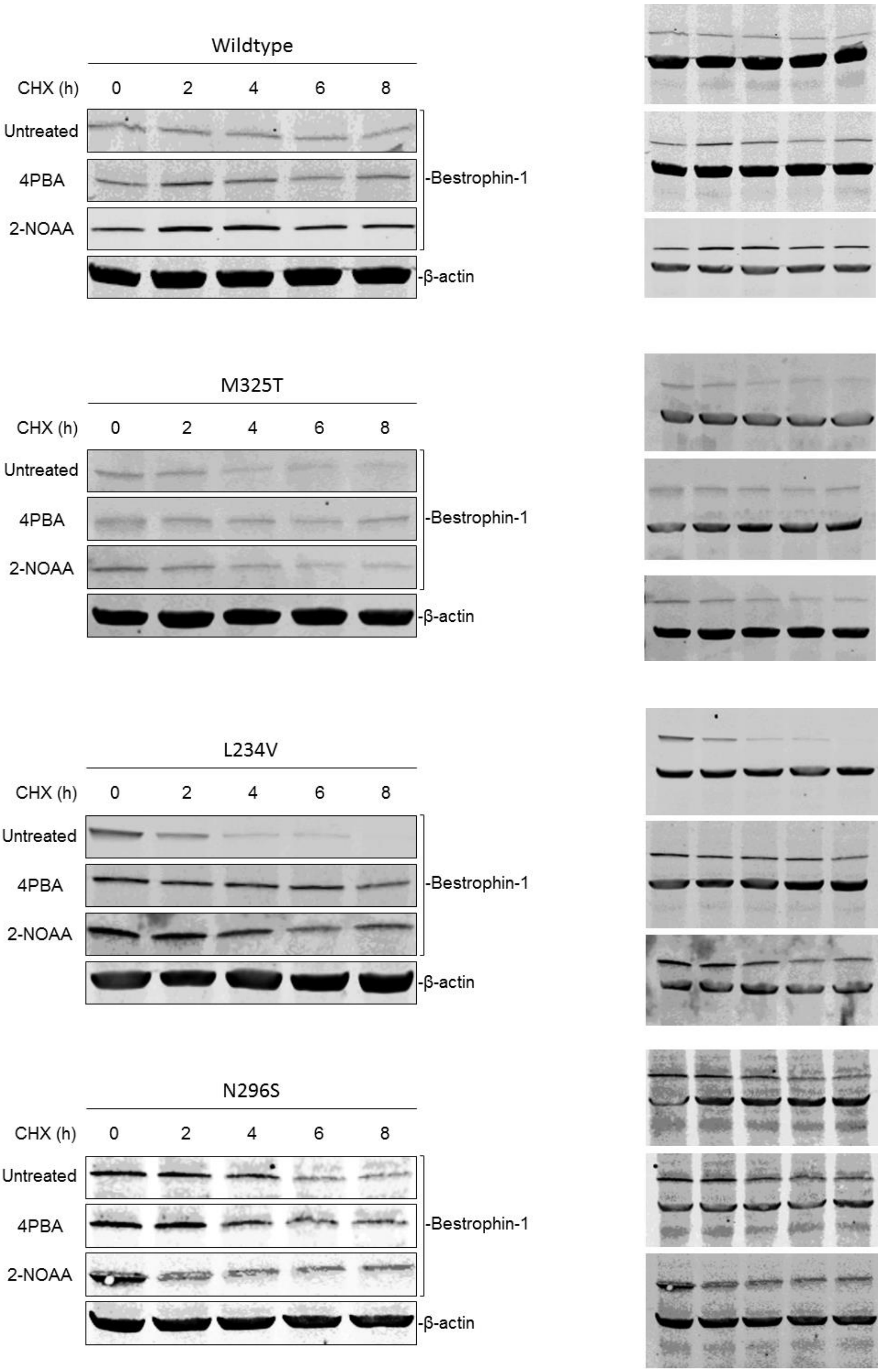

Fig. S2. Western blot results of stable MDCKII cells treated by CHX for 0-8 h. The original blots with loading control were shown on the right, corresponding with each blot presented on the left.

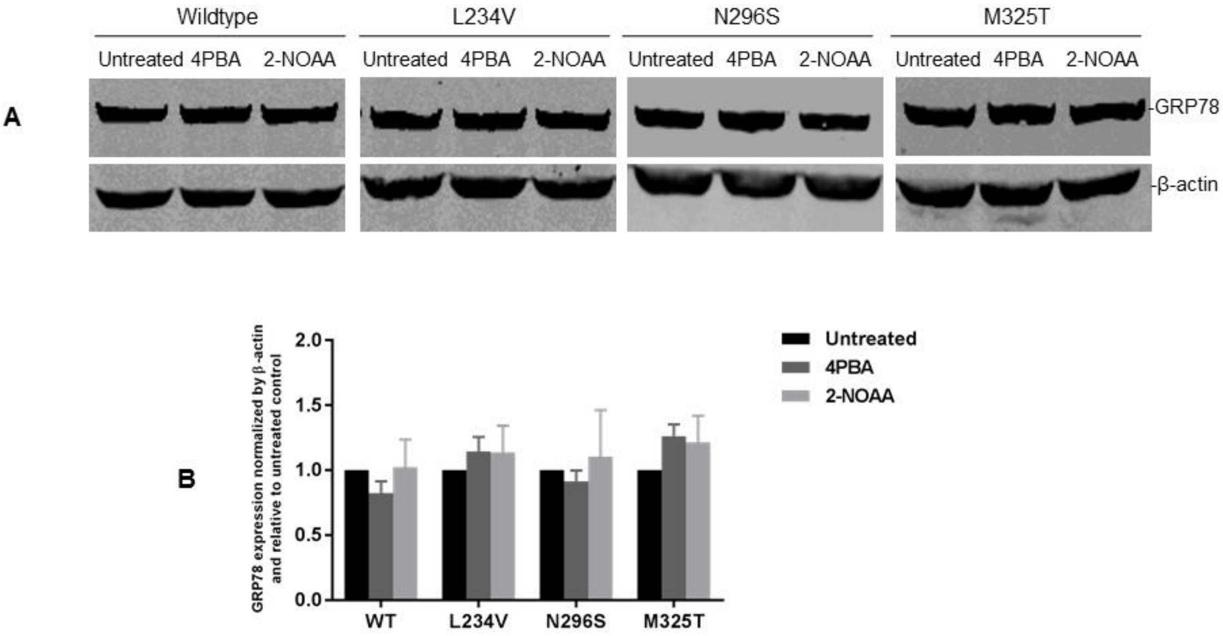

Fig. S3. Effect of 4PBA and 2-NOAA on ER-stress. (**A**) Western blot results showing the effect of 4PBA and 2-NOAA on the expression of the ER-stress marker 78-kDa glucose-regulated protein (GRP78) in MDCKII cells expressing wildtype or mutant bestrophin-1. (**B**) Quantification of western blot data from at least 3 independent experiments. The expression of GRP78 is normalised to the loading control (β-actin) and relative to untreated group that is taken as 1 in the graph. No significant difference was seen with one-way ANOVA analysis.

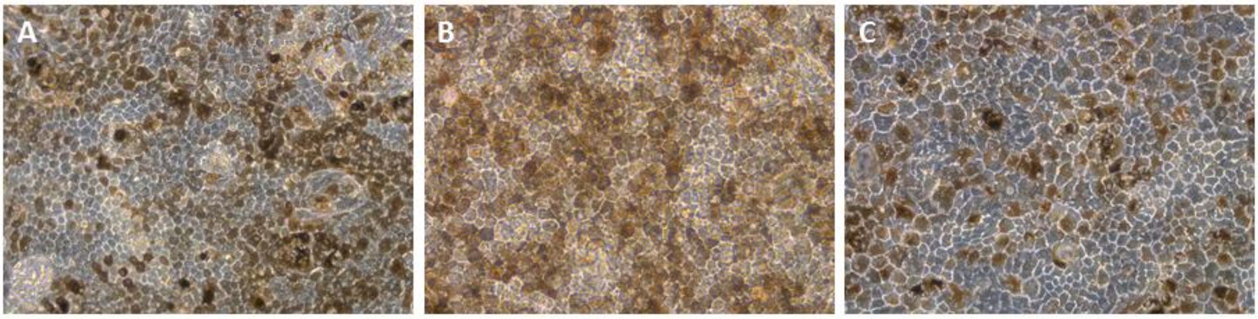

Fig. S4. iPSC-RPE. (**A**) Wildtype iPSC-RPE (passage 4); (**B**) Bestrophin-1 p.R255Q iPSC-RPE (passage 4). (**C**) Bestrophin-1 p.K30R iPSC-RPE (passage 4).

Supplementary Table

Table S1. List of antibodies used in this study

Primary antibodies

Target	Host species	Dilution	Supplier	Catalogue No.
**Bestrophin-1**	Mouse	1:4000 (WB)<br>1:500 (IF)	Abcam	Ab2182
**Beta actin**	Rabbit	1:3000 (WB)	Abcam	Ab8227
**MCT-1**	Rabbit	1:500 (IF)	Abcam	Ab85021
**GRP78**	Mouse	1:1000 (WB)	Abcam	Ab12223

Secondary antibodies

Antibody	Target	Host species	Dilution	Supplier	Catalogue No.
**IRDye® 680RD**	Mouse IgG	Donkey	1:5000 (WB)	LI-COR	925-68072
**IRDye® 800CW**	Rabbit IgG	Donkey	1:5000 (WB)	LI-COR	926-32213
**Alexa Fluor™ 568**	Mouse IgG1	Goat	1:500 (IF)	Life technologies	A21124
**Alexa Fluor™ 488**	Rabbit IgG	Donkey	1:500 (IF)	Life technologies	A21206

WB=western blotting; IF=Immunofluorescence

References:

1. Yang W, Mills JA, Sullivan S, Liu Y, French DL, Gadue P. iPSC Reprogramming from Human Peripheral Blood Using Sendai Virus Mediated Gene Transfer. In: *StemBook.* Cambridge (MA)2008.
2. Leach LL, Croze RH, Hu Q, et al. Induced Pluripotent Stem Cell-Derived Retinal Pigmented Epithelium: A Comparative Study Between Cell Lines and Differentiation Methods. *J Ocul Pharmacol Ther.* 2016;32(5):317-330.
